# High Carbohydrate Antigen 19-9 Levels Indicate Poor Prognosis of Upper Tract Urothelial Carcinoma

**DOI:** 10.3389/fonc.2022.858813

**Published:** 2022-07-14

**Authors:** Seung-hwan Jeong, Jang Hee Han, Chang Wook Jeong, Hyeon Hoe Kim, Cheol Kwak, Hyeong Dong Yuk, Ja Hyeon Ku

**Affiliations:** ^1^ Department of Urology, Seoul National University Hospital, Seoul, South Korea; ^2^ Department of Urology, Seoul National University College of Medicine, Seoul, South Korea

**Keywords:** CA 19-9, UTUC, prognosis, survival, metastasis

## Abstract

Upper tract urothelial carcinoma (UTUC) occurs in urothelial cells from the kidney and the ureters. Carbohydrate antigen 19-9 (CA 19-9) is a tumor marker for pancreatic and gastrointestinal cancers, and its high levels are associated with poor prognosis in bladder cancer. In this study, prospective patients enrolled in the registry of Seoul National University were retrospectively examined to determine the clinical significance of CA 19-9 in UTUC. In 227 patients, high serum CA 19-9 levels reflected a high tumor burden represented by high T and N stages, leading to adverse prognosis in metastasis-free or overall survival. Subsequently, propensity score matching analysis showed that the CA 19-9 level is an independent prognostic factor of UTUC.

## Introduction

Urothelial carcinoma arises from epithelial cells lining the urinary system. Most urothelial carcinomas occur in the urinary bladder, whereas upper tract urothelial carcinoma (UTUC), involving the renal calyx, pelvis, and ureter, accounts for 5%–10% of urothelial carcinomas (1, 2). The prognosis of UTUC depends on the T stage, which shows a 5-year survival rate from 90.2% to 18.5% through stages T1 to T4 (3). Risk classification stratifies UTUC as low- or high-risk, with low-risk cases allowing kidney-sparing surgeries, such as segmental ureterectomy and endoscopic ablation (4). In contrast, definitive treatment with nephroureterectomy is required for high-risk patients with adverse features. In addition, perioperative chemotherapy provides benefits in overall survival (OS) and cancer-specific survival with much concrete evidence in an adjuvant setting (5). The clinical staging of UTUC is restricted because of the pitfalls of computed tomography (CT) urography in discriminating between the T stages of carcinoma *in situ* and T2 (6). Thus, appropriate tools are required to evaluate the disease burden and to stratify risk classification.

Carbohydrate antigen 19-9 (CA 19-9) is a sialylated Lewis antigen. It is a tumor marker that predicts tumor stage, disease burden, and recurrence in pancreatic and gastrointestinal cancers (7–9). Although CA 19-9 is not a diagnostic marker in urothelial carcinoma, it is reportedly associated with the disease burden and aggressive features of bladder cancer, implying poor prognosis (10–12). In the present study, serum CA 19-9 levels in patients with UTUC were evaluated to reveal its clinical relevance implicating tumor burdens and clinical outcomes.

## Materials and Methods

### Study Subjects

The analyzed clinical data were of patients with UTUC enrolled in the Seoul National University Prospective Enrolled Registry for urothelial cancer from March 2016 to December 2020 with institutional review board approval (IRB No. 2201-032-1289) (13). From 420 patients, 227 patients whose preoperative serum CA 19-9 levels were measured were selected and stratified into low- (≤ 37 U/ml) and high-CA 19-9 (> 37 U/ml) level groups as normal value of CA 19-9 is considered to be lower than 37 U/ml (14). Preoperative and postoperative data, including the underlying disease, clinical and pathologic stage, and findings, were queried and compared.

### Statistical Analysis

Two-tailed t-tests were performed on parametric values, such as age, body mass index (BMI), and CA 19-9 level. The chi-square test was performed for categorical variables, including sex, underlying disease status, clinical and pathologic stage, hydronephrosis, perioperative chemotherapy, and tumor grade. Metastasis-free survival and OS were analyzed using Kaplan–Meier survival analysis, with the log-rank test for significance evaluation. To alleviate confounding effects derived from tumor burdens correlated with CA 19-9 levels, propensity score matching (PSM) was conducted to match pathologic T and N stages with a 1:4 ratio in both patient groups. Statistical analysis was performed using XLSTAT (version 2021.5-life sciences). Statistical significance was set at p < 0.05.

## Results

### CA 19-9 Is Related to High Tumor Burden

**Figure 1:**
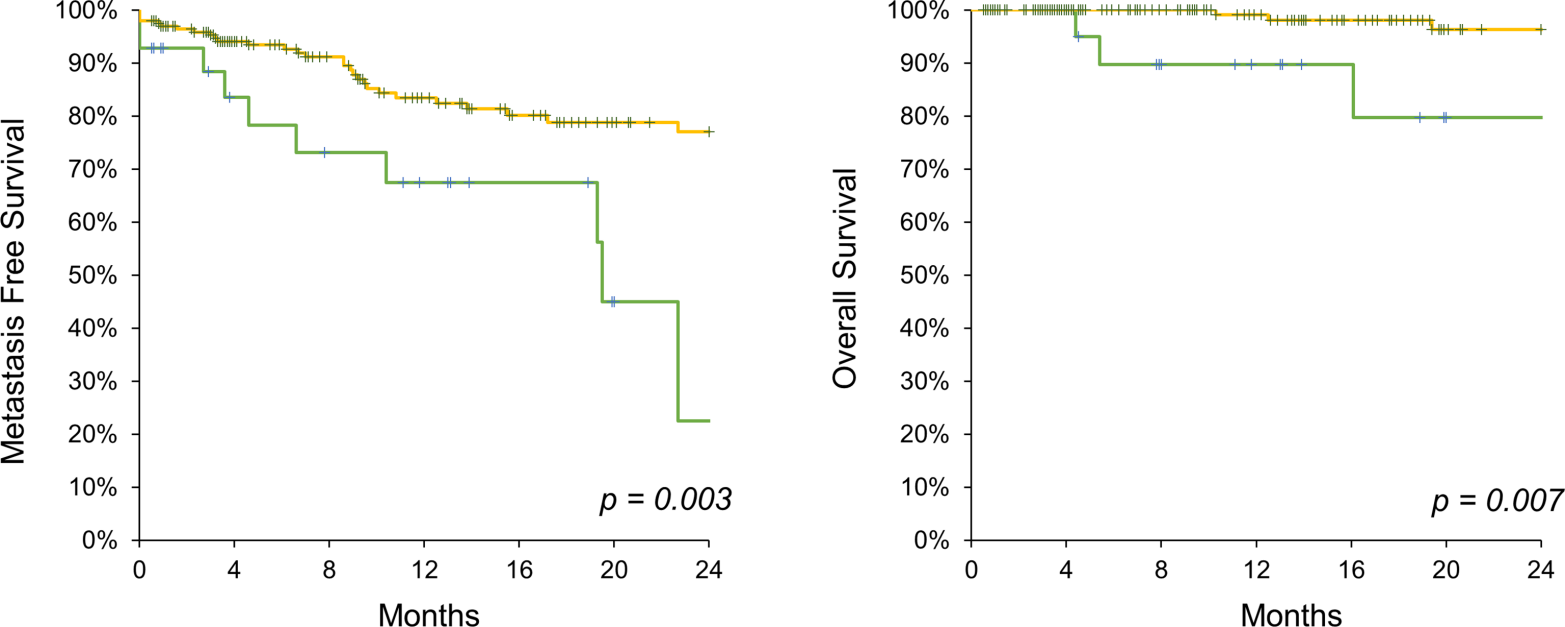
Kaplan-Meier analysis on metastasis free survival (left) and overall survival (right) comparing CA 19-9 high (green line) and low (yellow line) UTUC patients.

In a total of 227 patients, 199 and 28 patients were classified into low- and high-CA 19-9-level groups, respectively. The two groups were similar in terms of demographic findings, such as sex (male proportion of 71% vs. 57.1%, p = 0.126), age (70.4 vs. 71.8 years, p = 0.485), and BMI (24.7 vs. 24.1%, p = 0.388) (**Table 1**). Underlying diseases, including hypertension, diabetes mellitus, liver disease, and dyslipidemia, were also similar between the two groups. Cisplatin-based neoadjuvant chemotherapy was administered to 3.02% and 3.57% patients in the low- and high-CA 19-9-level groups, respectively (p = 0.873). All patients underwent nephroureterectomy *via* open, laparoscopic, or robotic procedures in similar proportions (p = 0.665). The clinical stage was significantly higher in the high-CA 19-9-level group, represented by 32.6% of the T3 or T4 stage population, compared with 21.1% in the low-CA 19-9-level group (p = 0.016). Accordingly, hydronephrosis was more prevalent in the high-CA 19-9-level group, without statistical significance. The pathologic T stage was higher in those with high CA 19-9 levels, with 69.9% of them having stage T3 or T4 tumors, compared to 36.7% of those with low CA 19-9 levels (p = 0.037). Furthermore, pathologic N1 or N2 stage was diagnosed in 21.5% of the patients in the high-CA 19-9 level group, which was higher than 4.0% in the low-CA 19-9 level group (p = 0.002). Cisplatin-based adjuvant chemotherapy was administered to similar proportion of patients in the two groups (22.6% vs. 28.6%, p = 0.485). Both the 2-year metastasis-free survival (77.0% vs. 22.5%, p = 0.003) and OS (96.4% vs. 79.8%, p = 0.007) rates were significantly higher in the low-CA 19-9-level group (**Figure 1**). COX regression analysis was performed to reveal factors associated with metastasis. Among the included variables, high CA19-9 level, high T stage and N stage were significantly associated with the risk of metastasis (**Table 2**).

**Table 1:** Characteristics of patients with low or high CA19-9 level.

	CA19-9 Low (n = 199)	CA19-9 High (n = 28)	*P* value
Sex Man Woman	142 (71.4%) 57 (28.6%)	16 (57.1%) 12 (42.9%)	0.126
Age	70.4	71.8	0.485
BMI	24.7	24.1	0.388
HTN	121 (60.8%)	15 (53.6%)	0.465
DM	66 (33.2%)	9 (32.1%)	0.914
Liver disease	18 (9.05%)	1 (3.57%)	0.327
Dyslipidemia	60 (30.2%)	9 (32.1%)	0.830
Clinical T stage Ta T1 T2 T3 T4	15 (7.54%) 64 (32.2%) 78 (39.2%) 42 (21.1%) 0	0 5 (17.9%) 14 (50%) 8 (28.6%) 1 (3.6%)	0.016
Hydronephrosis	96 (48.2%)	18 (64.3%)	0.112
Neoadjuvant CTx	6 (3.02%)	1 (3.57%)	0.873
Operation Open Laparoscopic Robotic	82 (41.2%) 28 (14.1%) 89 (44.7%)	14 (50%) 3 (10.7%) 11 (38.3%)	0.665
Pathologic T stage Ta CIS T1 T2 T3 T4	45 (22.6%) 6 (3.0%) 52 (26.1%) 23 (11.6%) 72 (36.2%) 1 (0.5%)	3 (10.7%) 0 4 (14.3%) 2 (7.1%) 18 (64.3%) 1 (3.6%)	0.037
Pathologic N stage Nx N0 N1 N2	164 (82.4%) 27 (13.6%) 1 (0.5%) 7 (3.5%)	16 (57.1%) 6 (21.4%) 1 (3.6%) 5 (17.9%)	0.002
Histologic Grade Low grade High grade	166 (83.4%) 33 (16.6%)	24 (85.7%) 4 (14.3%)	0.758
Adjuvant CTx	45 (22.6%)	8 (28.6%)	0.485
CA 19-9 (U/mL)	7.73	255.96	< 0.0001

**Table 2:** COX regression analysis for metastasis.

	HR (95% CI)	*P* value
Age	1.02 (0.981 - 1.057)	0.337
CA 19-9 (U/ml)	1.001 (1.000 – 1.003)	0.036
Hydronephrosis	1.36 (0.709 – 2.601)	0.357
Histologic grade Low grade High grade	Reference 0.232 (0.063 – 1.96)	Reference 0.232
Pathologic T stage Ta CIS T1, T2 T3, T4	Reference 1.87E-7 (0.000 – 0.000) 6.89 (0.87 – 54.679) 30.36 (3.661 – 251.81)	Reference 0.995 0.068 0.002
Pathologic N stage N0 Nx N1 N2	Reference 0.752 (0.328 – 1.725) 0.67 (0.081 – 5.684) 4.63 (1.529 – 14.016)	Reference 0.501 0.719 0.007

### PSM Revealed CA 19-9 as an Independent Factor for Tumor Burden and Prognosis

**Figure 2:**
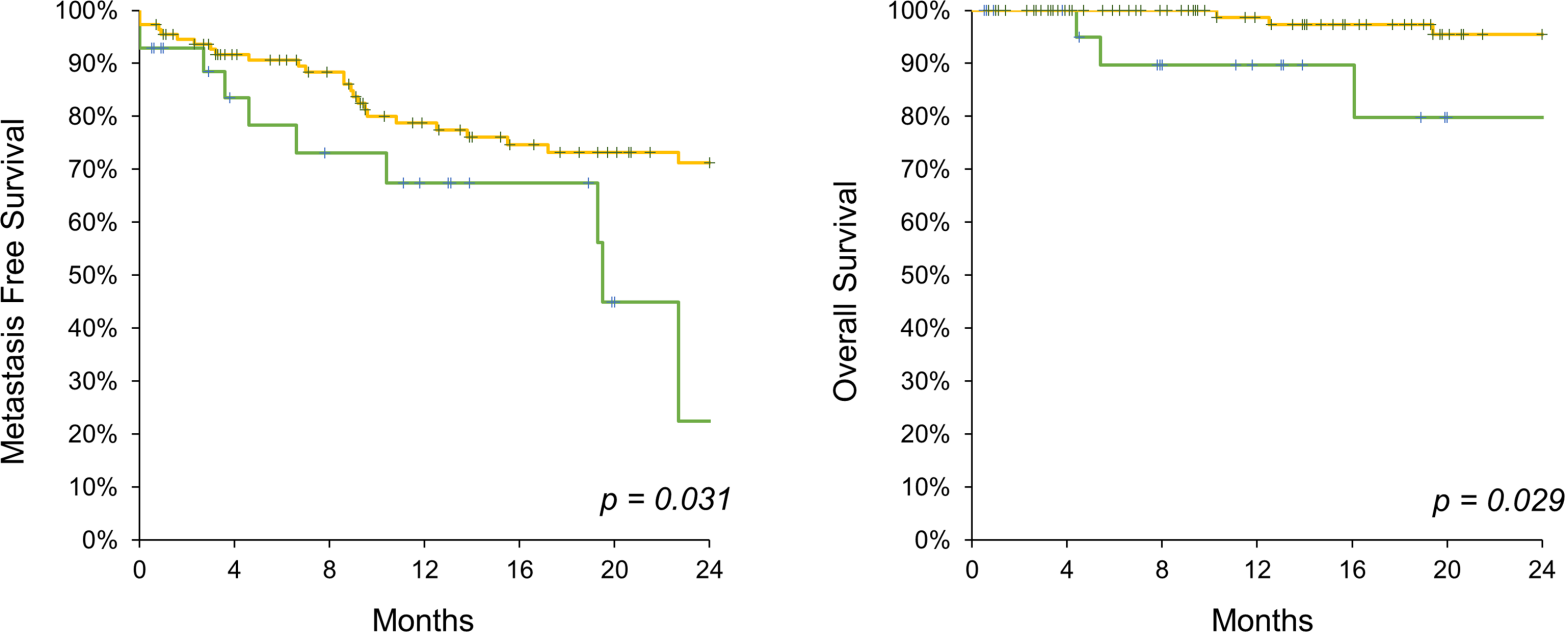
Kaplan-Meier analysis on metastasis free survival (left) and overall survival (right) comparing CA 19-9 high (green line) and low (yellow line) UTUC patients following propensity score matching.

In the high-CA 19-9-level group, the tumor burden was higher, leading to poor prognosis. To identify CA 19-9 as an independent prognostic factor, PSM analysis was performed for pathological T and N stages. In the PSM cases, demographic findings and underlying diseases remained relatively different between the two groups (**Table 3**). Clinical stage did not differ between the two groups (p = 0.123), which was reflected in the incidence of hydronephrosis (p = 0.269). Neoadjuvant or adjuvant chemotherapy was administered to similar proportion of patients in both groups. The pathologic T stage was matched in similar proportions between the two groups, represented by 64.3% and 67.9% of patients with T3 or higher stage in the low- and high-CA 19-9-level groups, respectively (p = 0.904). The pathologic N stage tended to be higher in the high-CA 19-9 level group, without statistical significance (p = 0.13). Interestingly, in the PSM analysis, the two-year metastasis free survival (71.2% vs. 22.5%, p = 0.031) and OS (95.4% vs. 79.8%, p = 0.029) rates were significantly higher in the low-CA 19-9-level group (**Figure 2**).

**Table 3:** Characteristics of propensity score matched patients.

	CA19-9 Low (n = 112)	CA19-9 High (n = 28)	*P* value
Sex Man Woman	81 (72.3%) 31 (27.7%)	16 (57.1%) 12 (42.9%)	0.119
Age	70.9	71.8	0.667
BMI	24.6	24.1	0.458
HTN	63 (56.3%)	15 (53.6%)	0.799
DM	39 (34.8%)	9 (32.1%)	0.789
Liver disease	12 (10.7%)	1 (3.6%)	0.244
Dyslipidemia	28 (25%)	9 (32.1%)	0.443
Clinical stage Ta T1 T2 T3 T4	9 (8%) 27 (24.1%) 44 (39.3%) 32 (28.6%) 0	0 5 (17.9%) 14 (50%) 8 (28.6%) 1 (3.6%)	0.123
Hydronephrosis	59 (52.7%)	18 (64.3%)	0.269
Neoadjuvant CTx	4 (3.57%)	1 (3.57%)	1.0
Operation Open Laparoscopic Robotic	63 (56.3%) 10 (8.9%) 38 (34.8%)	14 (50%) 3 (10.7%) 11 (38.3%)	0.835
Pathologic T stage Ta CIS T1 T2 T3 T4	11 (9.8%) 1 (0.9%) 17 (15.2%) 11 (9.8%) 71 (63.4%) 1 (0.9%)	3 (10.7%) 0 4 (14.3%) 2 (7.1%) 18 (64.3%) 1 (3.6%)	0.904
Pathologic N stage Nx N0 N1 N2	83 (74.1%) 21 (18.8%) 1 (0.9%) 7 (6.3%)	16 (57.1%) 6 (21.4%) 1 (3.6%) 5 (17.9%)	0.130
Histologic Grade Low grade High grade	104 (92.9%) 8 (7.1%)	24 (85.7%) 4 (14.3%)	0.227
Adjuvant CTx	43 (38.4%)	8 (28.6%)	0.334
CA19-9 (U/mL)	7.37	255.96	< 0.0001

## Discussion

In bladder cancer, CA 19-9 is associated with adverse pathologic stages, characterized by muscular layer invasion and metastasis, thereby leading to poorer survival rates in bladder cancer patients with high CA 19-9 levels (12, 15). Furthermore, accumulating data suggest that urothelial cancers might produce CA19-9 to reflect tumor aggressiveness and tumor burdens (10, 12, 16).

However, there have been no reports evaluating the prognostic value of CA 19-9 for UTUC. In the present study, CA 19-9 was associated with a high tumor burden represented by higher T and N stages, and led to worse outcomes in metastasis-free survival and OS. CA 19-9 is highly expressed in the serum of patients with pancreatic or colon cancer (17). In pancreatic cancer, CA 19-9 is a useful diagnostic and prognostic marker for evaluating the tumor stage, treatment response, and OS. Similar to the study on UTUC, preoperative CA 19-9 levels are associated with tumor resectability and pancreatic cancer stage. A decrease in CA 19-9 levels after surgery reflects favorable survival outcomes, and elevated CA 19-9 levels imply worse survival outcomes (18). In addition, the CA 19-9 level is useful for evaluating disease progression or remission in response to treatment (19, 20). This study investigated only preoperative CA 19-9 levels, but serial measurements following treatment would be valuable in predicting prognostic outcomes. The diagnostic value of CA 19-9 is disappointing because of high false-positive rates in normal conditions and other diseases, such as liver cirrhosis, pancreatitis, and benign gastrointestinal diseases (21). However, in discriminating between benign and malignant pancreatic nodules, it is valuable, with a specificity of 90%.

In this study, PSM analysis was conducted to mitigate adverse features, such as T and N stages, reflecting tumor burden, which is associated with poor outcomes. PSM corrected the imbalance between low- and high-CA 19-9 level groups regarding T and N stages, reflecting tumor burden. Interestingly, after PSM analysis, high CA 19-9 levels indicated worse prognosis, thereby affirming CA 19-9 as an independent prognostic marker, not only based on tumor burden but also its aggressiveness. Similar findings have been reported in pancreatic cancer, providing worse prognosis in multivariate analysis of CA 19-9 level, tumor grade, and tumor size (22). Furthermore, in colorectal cancer, high CA 19-9 levels are related to poor oncologic outcomes, including OS and disease-free survival on PSM analysis (23).

This study is limited by the fact that it had a relatively small sample size and a retrospective study design. However, this report is valuable, considering the low incidence of UTUC with concomitant measurement of CA 19-9 and data queries from a prospective patient enrollment system. Moreover, monitoring CA 19-9 levels may provide preoperative risk classification and facilitate strategic follow-up and adjuvant treatment. Thus, further studies are required to include a larger number of patients and serial follow-up of CA 19-9 in the treatment course.

## Data Availability Statement

The raw data supporting the conclusions of this article will be made available by the authors, without undue reservation.

## Ethics Statement

The studies involving human participants were reviewed and approved by Institutional review board of Seoul National University Hospital. Written informed consent for participation was not required for this study in accordance with the national legislation and the institutional requirements.

## Author Contributions

Conceptualization: S-HJ. Data collection: S-HJ, JH, HY, CJ, HK, JK, CK. Data analysis: S-HJ, HY, JK. Data visualization: S-HJ. Data interpretation: S-HJ, JH, HY, CJ, HK, CK, JK. Manuscript writing: S-HJ. Supervision: S-HJ, HY, JK. All authors contributed to the article and approved the submitted version.

## Funding

This research was supported by a Basic Science Research Program through National Research Foundation of Korea (NRF), funded by the Ministry of Education (NRF-2018R1D1A1B07041191) and by Seoul National University Hospital (0320202190).

## Conflict of Interest

The authors declare that the research was conducted in the absence of any commercial or financial relationships that could be construed as a potential conflict of interest.

## Publisher’s Note

All claims expressed in this article are solely those of the authors and do not necessarily represent those of their affiliated organizations, or those of the publisher, the editors and the reviewers. Any product that may be evaluated in this article, or claim that may be made by its manufacturer, is not guaranteed or endorsed by the publisher.
